# Can presepsin uniformly respond to various pathogens? - an in vitro assay of new sepsis marker -

**DOI:** 10.1186/s12865-020-00362-z

**Published:** 2020-06-05

**Authors:** Yusuke Koizumi, Daisuke Sakanashi, Tetsuo Mohri, Hiroki Watanabe, Arufumi Shiota, Nobuhiro Asai, Hideo Kato, Mao Hagihara, Kenta Murotani, Yuka Yamagishi, Hiroyuki Suematsu, Hiroshige Mikamo

**Affiliations:** 1 Department of Clinical Infectious Diseases, Aichi Medical University Hospital, 1-1 Yazakokarimata, Nagakute, Aichi 480-1195 Japan; 2 Department of Infection Control, Aichi Medical University Hospital, Nagakute, Aichi, Japan; 3 Biostatistics center, Graduate School of Medicine, Kurume University, Kurume, Fukuoka, Japan

**Keywords:** Presepsin, CD14, Bacteremia, Gram-positive bacteria, Gram-negative bacteria, Lipopolysaccharide, In vitro assay

## Abstract

**Background:**

Presepsin (soluble CD14 subtype) is a novel biomarker of sepsis used for early diagnosis. Originally, CD14 is known as the pattern recognition receptor for the lipopolysaccharide (LPS)/LBP complexes, and the diagnostic value of this molecule for gram-positive bacteria, which contain less amount of LPS, is uncertain. To confirm its effectiveness in the diagnosis of bacteremia caused by gram positive bacteria, and to evaluate the interspecies difference of presepsin production against various bacterial strains, we conducted an in vitro assay to evaluate presepsin levels in response to five Gram negative and four Gram positive bacteria.

**Results:**

Whole blood was yielded from five healthy volunteers and co-cultured with bacterial strains at 37 °C for 4 h. Twenty seven clinical isolates of nine species (*E. coli*, *K. pneumoniae*, *E. cloacae*, *P. aeruginosa*, *S. maltophilia*, *S. aureus*, *S. pyogenes*, *B. cereus*, and *C. striatum*) derived from blood cultures of non-neutropenic bacteremia patients were used. Culture supernatants were harvested and presepsin levels were measured. The presepsin level in the gram-negative bacteria 273 (218–352) pg/mL was significantly higher than in the gram-positive bacteria 200 (143–275) pg/mL (*p* = 0.0002). The presepsin levels were significantly lower in *C. striatum*, in comparison to other bacteria, and *S. pyogenes* showed similar results. And the presepsin levels in *P. aeruginosa* were significantly lower compared to *E. cloacae*, *K. pneumoniae*, and *S. aureus*.

**Conclusions:**

Presepsin production can also be evoked by gram-positive bacteria, and interspecies differences of presepsin response may exist, which should be considered in the diagnosis of sepsis, especially in certain gram-positive bacteremia such as *S. pyogenes* or *C. striatum*.

## Background

Presepsin (soluble CD14 subtype) is a novel biomarker of sepsis [1]. The plasma presepsin level rises as early as within 2 h of inflammation onset, which is even earlier than C-reactive protein (CRP), procalcitonin, or interleukin-6 (IL-6) [2–4]. With a high specificity for bacterial infections, presepsin has proved useful in early diagnosis and is a prognostic marker of severe sepsis [3–8]. In recent years, the clinical importance of this molecule has been intensively discussed in various categories of infection such as surgery [9], burn [10], neonatal sepsis [11], and febrile neutropenia [12, 13]. Moreover, recent meta-analyses showed its efficacy in the diagnosis of sepsis [14, 15].

CD14 is the receptor for lipopolysaccharide (LPS) / LPS – binding protein (LPS/LBP) complexes [16] and is released in the early phase of infection. As LPS represents a characteristic attribute of gram-negative bacteria, the diagnostic value of this molecule for gram-positive bacteria, which contain less LPS, is uncertain.

In this study, we conducted a simple in vitro assay to evaluate presepsin levels in response to various gram-negative and gram-positive bacterial strains. Although other factors might influence the presepsin altitude in real septic patient’s environment, we tried to clarify the interspecies differences in response to various pathogens with this in vitro assay.

## Results

**Fig. 1:**
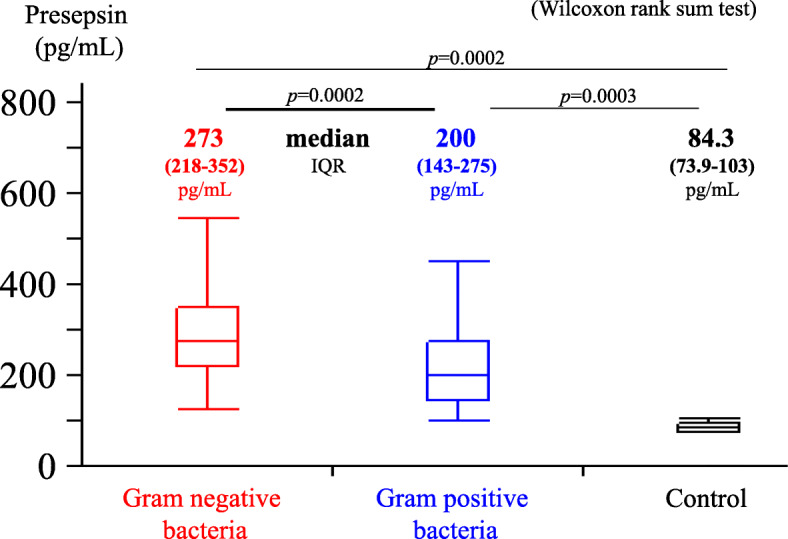
Comparison of the presepsin levels between gram-negative and gram-positive bacteria. The presepsin levels after 4 h of incubation were significantly higher in gram-negative bacteria (median 273, [IQR, 218–352]) pg/mL) than in gram-positive bacteria (median 200, [IQR,143–275]) pg/mL, *p* = 0.0002, Wilcoxon rank sum test). The presepsin values in control group was 84.3(73.9–103) pg/mL and significantly lower than both gram-negative and gram-positive bacteria (*p* = 0.0002 and 0.0003, respectively, Wilcoxon rank sum test). IQR, interquartile range. The vertical line within the box represents the median sample value. The ends of the box represent the 3rd and 1st quartile, respectively. The whiskers denote maximal and minimal values (not including outliers)

Figure 1 shows the comparison of presepsin levels between the gram-positive and gram-negative bacteria. The presepsin level in the gram-negative bacteria 273 (median, IQR = 218–352) pg/mL was significantly higher than in the gram-positive bacteria 200 (143–275) pg/mL (*p* = 0.0002, Wilcoxon rank sum test). The presepsin values in control group was 84.3 (73.9–103) pg/mL.

**Fig. 2:**
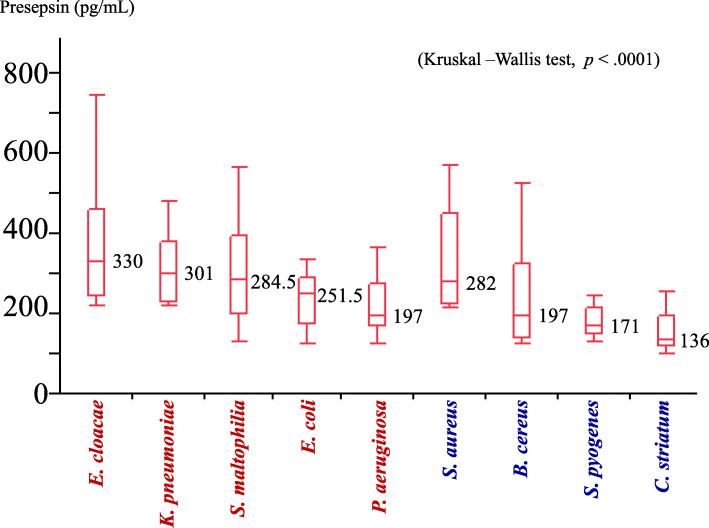
The species-specific presepsin levels. The presepsin levels after 4 h of incubation are shown in descending order of median values. Gram negative bacteria are on the left, and gram positive bacteria are on the right. The median values (interquartile ranges) of the gram-negative bacteria were, *E. cloacae* 330 (246–462) pg/mL, *K. pneumoniae* 301 (229–378) pg/mL, *S. maltophilia* 284.5 (202.25–396) pg/mL, *E. coli* 251.5 (175–289.25) pg/mL, and *P. aeruginosa* 197 (169–274) pg/mL. The median values of the gram-positive bacteria were, *S. aureus* 282 (225–452) pg/mL, *B. cereus* 197 (140–324) pg/mL, *S. pyogenes* 171 (150–213) pg/mL, and *C. striatum* 136 (119–193) pg/mL. The vertical line within the box represents the median sample value. The ends of the box represent the 3rd and 1st quartile, respectively. The whiskers denote maximal and minimal values (not including outliers)

Figure 2 shows the comparison of the presepsin released in the assay of each species. The strains were sorted in the descending order of median values of the amount of presepsin released. The median (IQR) of the presepsin levels in gram-negative bacteria were *Enterobacter cloacae* 330 (246–462) pg/mL, *Klebsiella. pneumoniae* 301 (229–378) pg/mL, *Stenotrophomonas maltophilia* 284.5 (202.25–396) pg/mL, *Escherichia coli* 251.5 (175–289.25) pg/mL, and *Pseudomonas aeruginosa* 197 (169–274) pg/mL.

The presepsin levels in the gram-positive bacteria were *Staphylococcus aureus* 282 (225–452) pg/mL, *Bacillus cereus* 197 (140–324) pg/mL, *Streptococcus pyogenes* 171 (150–213) pg/mL, and *Corynebacterium striatum* 136 (119–193) pg/mL.

The Steel-Dwass test revealed that the presepsin levels were significantly lower in *C. striatum*, in comparison to *E. cloacae* (*p* = 0.0003), *K. pneumoniae* (*p* = 0.0005), *S. aureus* (*p* = 0.0005), *S. maltophilia* (*p* = 0.018), and *E. coli* (*p* = 0.047), respectively.

The presepsin levels were also significantly lower in *S. pyogenes* in comparison to *E. cloacae* (*p* = 0.0003), *K. pneumoniae* (*p* = 0.0006), and *S. aureus* (*p* = 0.0007), respectively.

The presepsin levels in *P. aeruginosa* were significantly lower compared to *E. cloacae* (*p* = 0.013).

## Discussion

Presepsin is a subtype of the N-terminal fragment of soluble CD14 with a molecular weight of 13 kDa. CD14 is known as the pattern recognition receptor (PRR) for the LPS-LBP complexes expressed on the cell membrane of the phagocytes. During phagocytosis, the CD14 molecule is internalized with the bacteria and cleaved into presepsin after phagolysosomal processing, and released into the plasma [1, 4, 5, 17].

Previous studies had focused on the clinical aspects of this molecule, such as sensitivity, specificity or positive predictive value in the diagnosis of infectious diseases. For example, higher presepsin level is indicative of severe sepsis and is also associated with poor prognosis [3–8, 14, 15]. In those studies, the species of the bacteria causing sepsis were not considered in detail.

Because presepsin derives from CD14, one of the major PRRs, the degree of its response is presumed to reflect the surface structure of the confronting pathogen. Since LPS is specific for gram-negative bacteria, we attempted to evaluate the response of this molecule towards gram-positive bacteria. This is important because the existence of an interspecies difference might affect the reliability of presepsin as an early marker of infectious diseases.

Thus, we designed an in vitro assay to evaluate the presepsin levels in response to various strains including five gram-negative and four gram-positive bacteria. The assay system mimics the situation in bacteremia, reflecting the immediate phagocyte-bacterial interaction with the help of other circulating blood cells, plasma proteins or the complement system. Generally, the host-microbe interaction is difficult to evaluate because there are cascades of complex intervening inflammatory responses. However, unlike other assays, our system involves minimal requirements of host-microbe immunity. Therefore, evaluations in a direct and in a real time manner can be achieved.

Our results suggested that presepsin production can be also evoked by gram-positive bacteria. However, presepsin levels in response to the gram-positive bacteria were lower than those of gram-negative bacteria, as expected because LPS is a characteristic attribute of gram-negative bacteria. This trend was prominent in *C. striatum* and *S. pyogenes*. But some gram-positive organisms like *S. aureus* evoked relatively high levels of presepsin and gram-negative bacteria like *P. aeruginosa* evoked lower levels. This could probably be due to the difference in the immunogenicity or phagocytosis evocativenesss (sensitivity) among the species. The association of CD14 ligands, other than LPS, might influence its production. Or, the low presepsin level in response to *S. pyogenes* might be partly explained by phagocytosis escaping mechanisms [18]. Further analyses of the membrane structures or other factors affecting the levels of presepsin release should be carried out to elucidate the reasons for such a response.

This study has a few limitations. First, the host immune profile was not analyzed in detail, except that the number of neutrophils and the baseline presepsin levels were confirmed to be equivalent. We cannot exclude the influence of other immunological factors, such as lymphocyte subsets, complement, or cytokines. Second, we only used clinical isolates. We selected typical non-neutropenic bacteremia cases as a source of the bacterial strains. The cases showed bacteremia in the absence of neutropenia, immunosuppressants, and mechanical obstructions, which indicated that they are virulent enough to cause bacteremia in the general population. We intended to reproduce bacteremia in vitro with real world strains; however, whether they can be considered representative strains is debatable. All the species were correctly identified by biochemical analysis and confirmed by MALDI-TOF-MS. Moreover, to avoid potential strain bias, we integrated the data of 3 strains for one species. Third, the presepsin levels observed in this study cannot be applied in clinical settings. The values discussed here are a relative comparison among the species and do not reflect the cut-off values of either sepsis or the septic shock diagnostic criteria.

Taking the strengths and limitations of this study into consideration, our result is still meaningful because our assay mimics the clinical situation of bacteremia in which bacteria encounters immune cells in the blood, and we can directly evaluate the total immune response. Our experiment also warrants the need to consider the interspecies difference when presepsin is used as a diagnostic tool in sepsis.

## Conclusions

In conclusion, using a simple in vitro presepsin releasing assay, we found that presepsin production can also be evoked by gram-positive bacteria, though the levels were significantly lower in comparison to the gram-negative bacteria. We also found interspecies differences in the presepsin response levels, which might be important in the diagnosis of infectious diseases caused by certain gram-positive bacteria, such as *S. pyogenes* or *C. striatum*. To our knowledge, this is the first report using an in vitro presepsin assay, and focusing on the difference of its responsiveness as a PRR against various bacterial species. Our experiment warrants the need to consider the interspecies difference when presepsin is used as a diagnostic tool in sepsis caused by certain gram-positive bacteria, such as *S. pyogenes* or *C. striatum*.

## Methods

The system is a co-culture assay using whole blood and the bacterial strains obtained from blood culture.

### Whole blood preparation

Venous blood samples were drawn from five healthy volunteers. All the participants had normal renal and hepatic function, normal leukocyte count, and no previous medical history. After phlebotomy, whole blood was immediately heparinized and prepared for the co-culture assay.

### Bacterial strains

We selected five gram-negative bacteria (*Escherichia coli, Klebsiella pneumoniae, Enterobacter cloacae, Pseudomonas aeruginosa,* and *Stenotrophomonas maltophilia*), and four gram-positive bacteria (*Staphylococcus aureus, Streptococcus pyogenes, Bacillus cereus,* and *Corynebacterium striatum*); three strains per species were examined. Twenty seven clinical isolates were obtained from the blood cultures of 27 different bacteremic patients, all of them demonstrating septic shock and/or disseminated intravascular coagulation at the time of blood culture. They neither had neutropenia, immunosuppression, nor mechanical obstructions in urinary, biliary, or gastrointestinal tracts.

The strains were identified by conventional biochemical methods and were confirmed by matrix-assisted laser desorption ionization-time of flight mass spectrometry (MALDI-TOF/MS) (Biotyper ver.3.1, Burker Daltonics, Bremen, Germany).

### Co-culture & measurement of presepsin level

As written above, fifteen co-cultures per each species (five volunteers for three strains) were examined. The bacterial strains were recovered from the deep freezer and sub-cultured in blood agar plates before use. Bacterial counts were determined by colorimetric analysis, and the concentrations were adjusted to obtain 2 × 10^6^ CFUs in 40 μL of saline.

The bacterial suspension and 400 μL of the whole blood were mixed in the microplates. The mixture was incubated at 37 °C for 4 h with shaking and culture supernatant was harvested. Presepsin measurement was made by a rapid chemiluminescent enzyme immunoassay on the fully automated PATHFAST® immunoanalyzer (Mitsubishi Chemical Medience Corporation, Tokyo, Japan) [19].

### Statistical analysis

The data are represented as median (interquartile range; IQR). In all analyses, *p* < 0.05 was considered statistically significant. Wilcoxon rank sum test was performed for comparisons of two independent groups of sampled data.

The Kruskal–Wallis test and Steel-Dwass test was performed to compare independent samples of equal or different sample sizes. We analyzed the data using the statistical software JMP® 10 (SAS Institute Inc., Cary, NC, USA).

## Data Availability

The datasets used and/or analyzed during the current study available from the corresponding author on reasonable request.

## Abbreviations

LPS: Lipopolysaccharide
LBP: LPS binding protein
MALDI-TOF/MS: Matrix-assisted laser desorption ionization-time of flight mass spectrometry
CFU: Colony forming unit
IQR: Interquartile range
PRR: Pattern recognition receptor,
